# Hype or hope? The potential of melatonin for *delirium* prevention in the intensive care unit

**DOI:** 10.62675/2965-2774.20260433

**Published:** 2026-05-22

**Authors:** Adriano José Maia Chaves, Viviane Cordeiro Veiga

**Affiliations:** 1 HCor-Hospital do Coração Research Institute São Paulo SP Brazil Research Institute, HCor-Hospital do Coração - São Paulo (SP), Brazil.; 2 BP A Beneficência Portuguesa de São Paulo São Paulo SP Brazil BP - A Beneficência Portuguesa de São Paulo - São Paulo (SP), Brazil.

## INTRODUCTION

*Delirium* is common in intensive care units (ICUs) and independently predicts outcomes that matter, such as higher costs, mortality, and long-term cognitive issues.^(1)^ Once overlooked, it is now recognized as a measurable sign of brain dysfunction and has been added to the Sequential Organ Failure Assessment (SOFA) 2.0 tool.^(2)^ However, it is worth highlighting that the SOFA 2.0 scores the need for drug treatment for *delirium* and not a specific diagnostic assessment of this condition, which raises questions on the validity of this new scoring system since no drug treatment has been widely recommended for *delirium*.^(2)^ *Delirium* may be linked with sleep, circadian rhythm, and critical illness, though the exact relationship is unclear. Intensive care unit-specific environment and critical illness can disrupt sleep and worsen neurocognitive symptoms. This viewpoint summarizes current research on *delirium's* association with sleep disturbances and briefly reviews evidence for melatonin use to prevent ICU *delirium*, while suggesting future directions.

## *DELIRIUM*: IMPACT ON OUTCOMES THAT MATTER

*Delirium* is a rapid-onset attention disturbance with cognitive impairment, unrelated to other neurocognitive disorders or altered arousal states. Risk factors include acute or severe illness, emergency surgery, polytrauma, sedatives (like benzodiazepines), anticholinergic drugs, mechanical ventilation, older age, and chronic diseases such as dementia or hypertension.^(1)^ Historically, *delirium* affected 60 - 80% of ventilated patients and 20 - 50% of other ICU patients, but improved diagnosis and management have lowered rates by about 25%.^(3)^ The 2018 clinical guidelines on pain, agitation/sedation, *delirium*, immobility, and sleep disruption recommend routine *delirium* monitoring with validated ICU tools, and the continuous optimization of the ICU care environment has been demonstrated to be effective in preventing *delirium*.^(4)^

*Delirium* in hospitalized patients is a strong independent predictor of mortality, increased hospital length of stay, readmissions, long-term cognitive impairment, and costs.^(5)^ A prospective cohort study of 275 adult medical and coronary ICUs demonstrated that after adjustment for multiple risk factors, *delirium* was independently associated with higher 6-month mortality, longer hospital stay, and a longer post-ICU stay.^(5)^ The true attributable risk of mortality to *delirium* was evaluated in other studies, which have confirmed this association.^(5)^

The Bringing to Light the Risk Factors and Incidence of Neuropsychological Dysfunction in ICU Survivors (BRAIN-ICU) study, a large, multicenter cohort of 821 adult ICU patients with respiratory failure and cardiogenic or septic shock, was conducted to determine the prevalence of long-term cognitive impairment following critical illness. At 3 months post-discharge, a neurocognitive score similar to Alzheimer's disease was found in 26% of patients, and a score similar to moderate traumatic brain injury was found in 40% of patients in both young and older patients. *Delirium* was the strongest independent predictor of cognitive impairment in this cohort.^(6)^

## SLEEP DISTURBANCES IN THE INTENSIVE CARE UNIT AS THE UNDERLYING BASIS FOR *DELIRIUM*

The sleep/wake cycle is regulated by two main processes: the circadian rhythm and sleep homeostasis.^(7)^ Sleep homeostasis, meanwhile, reflects the body's rising need for sleep during wakefulness, which lessens with sleep, and is influenced by factors such as sleep duration, quality, and previous wake timeawareness, and cognition. It is highly prevalent among critically ill patients and is associated with increased morbidity and mortality. A core domain of delirium is represented by behavioural disturbances in sleep-wake cycle probably related to circadian rhythm disruption. The relationship between sleep, circadian rhythm and intensive care unit (ICU).^(7)^

Sleep in the ICU is often characterized by subjective poor quality. Critically ill patients experience increases in stages 1 and 2 sleep with frequent arousals and awakenings. Further, they are less likely to transition into stage 3, slow-wave, or REM (Rapid Eye Movement) sleep, reducing the time spent in these physiological states.^(8)^ Roughly 50% of sleep in the ICU is shifted to daytime hours, with implications for rehabilitation. Sleep disruption in the ICU is linked to artificial light, noise, illness, and treatments affecting circadian rhythms. Poor sleep has also been associated with *delirium* and other outcomes, such as length of ICU stay and long-term cognitive impairment.^(9)^

Disruption of neural networks that regulate circadian rhythm is observed in critically ill patients, especially those with *delirium*. Choi et al. found altered functional connectivity between the posterior cingulate cortex and components of the ascending reticular activating system (ARAS) in 22 medical inpatients during *delirium*.^(10)^ Disrupted melatonin activity is linked to *delirium*. Studies found that critically ill patients exhibit impaired melatonin secretion in response to light, suggesting circadian clock dysregulation.^(11)^ Despite this, a direct causal relationship between melatonin changes and *delirium* remains unproven.

## USAGE OF MELATONIN FOR *DELIRIUM* PREVENTION AND THE CURRENT STATE-OF-THE-ART

In fact, melatonin is a pineal-derived hormone that is central to the regulation of circadian sleep-wake rhythms. It is not stored but is immediately released into the bloodstream and cerebrospinal fluid.^(7)^ The evidence of deficient melatonin levels/secretion in critically ill patients makes it theoretically reasonable to expect beneficial effects of melatonin to enhance sleep quality and possibly reduce *delirium* in ICU settings.

Although melatonin has shown promise for improving sleep quality, its effectiveness in preventing or treating ICU *delirium* remains uncertain due to conflicting study results. Recent systematic reviews and meta-analyses reported inconsistent outcomes and noted limitations, including small sample sizes, varying melatonin doses, and different *delirium* assessment methods.^(12–15)^ Also, previous trials examining the effects of melatonin on *delirium* incidence in the ICU reported discrepancies.^(16–18)^ Large trials, such as the Pro-MEDIC trial,^(17)^ reported no significant difference in the number of *delirium*-free days between the melatonin and placebo groups. The DEMEL trial, which randomized patients to low-dose melatonin (0.3mg/day), high-dose melatonin (3mg/day), or placebo for 14 days, similarly found no significant difference in *delirium* incidence between melatonin and placebo.^(18)^

However, other randomized clinical trials have reported positive findings for the use of melatonin to reduce *delirium* risk in the ICU, especially in postsurgical populations, such as those undergoing percutaneous coronary intervention.^(19)^

## CONCLUSIONS

**Figure 1:**
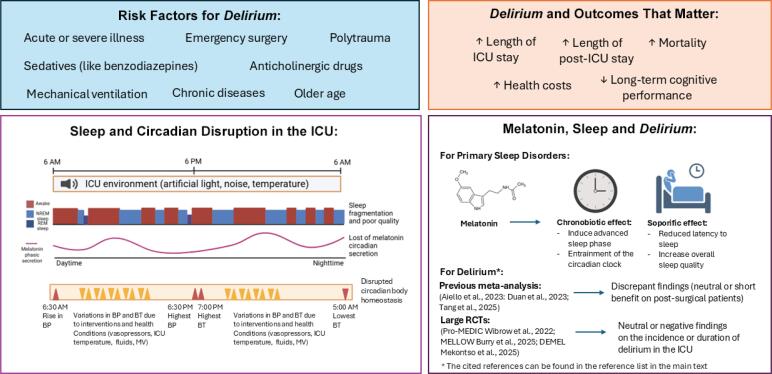
Key summary points on the sleep/circadian abnormalities in the intensive care unit, their association with *delirium*, and its impact on clinical outcomes that matter, and melatonin's therapeutic effects for sleep, and a summary of findings of its actions on *delirium* in the intensive care unit [based on previous meta-analysis^(13–15)^ and randomized clinical trials^(16–18)^].

Despite progress in understanding the sleep-wake cycle, gaps remain in understanding its links to circadian rhythm and *delirium* in ICU patients. Melatonin appears safe in the intensive care unit, but current randomized clinical trial evidence is inconsistent and does not support its routine use for *delirium*. While some data suggest benefits for certain patients, further studies are necessary (an panoramic view of the state of the art in the field, including the neurobiological rationale for the use of melatonin in the intensive care unit, as well as the main results of clinical trials and meta-analyses is shown in Figure 1). Advancing knowledge of sleep and circadian disruptions may pave the way for better pharmacological interventions to improve sleep quality and potentially reduce *delirium* in the intensive care unit.

ICU - intensive care unit; BP - blood pressure; BT - body temperature; MV - mechanical ventilation; RCT - randomized clinical trials.

Additionally, the bottom-left corner square represents the commonly observed circadian disturbances among intensive care unit patients. Notably, the presence of continuous external stimuli (e.g., artificial light, noise, and temperature), together with the patient's and illness characteristics, can induce poor sleep quality with marked sleep fragmentation, abnormal secretion of melatonin, and disrupted circadian body homeostasis in response to these signals, such as loss of circadian variations in blood pressure and body temperature.

## Data Availability

No datasets were generated or analyzed during the current study. This article is based on the critical appraisal and synthesis of previously published literature, all of which is cited in the reference list.
